# Agro-morphological and molecular characterization of Amaranthus genotypes

**DOI:** 10.1371/journal.pone.0328567

**Published:** 2025-09-23

**Authors:** Jonathan Siamey, Jacqueline Naalamle Amissah, Peter Amoako Ofori, Richard Adu Amoah, Eric Opoku Mensah, Daniel Ashie Kotey

**Affiliations:** 1 Plant Genetic Resources Research Institute, Bunso, Eastern Region, Ghana; 2 Department of Crop Science, University of Ghana, Legon, Greater Accra Region, Ghana; 3 Biotechnology Centre, University of Ghana, Legon, Greater Accra Region, Ghana; Nuclear Science and Technology Research Institute, IRAN, ISLAMIC REPUBLIC OF

## Abstract

Amaranth is a climate-resilient indigenous leafy vegetable that has the potential to contribute to global food security. Owing to its high protein, essential amino acid, mineral and vitamin contents, amaranth can thrive under diverse environmental conditions including marginal soils and drought-prone areas. This study characterized 21 Amaranth accessions using a combination of agro-morphological and molecular markers to identify promising genotypes for breeding programs. Characterization was carried out at two locations using a randomized complete block design (RCBD) with three replications. Six SSR and four ISSR primers were used to assess molecular diversity. The combined analysis of variance revealed significant differences in the majority of traits measured. Frequency distribution analysis revealed the predominance of many branches, green leaf pigmentation, lanceolate and elliptical leaf shapes, and smooth leaf vein prominence. Phylogenetic association analysis grouped the genotypes into three distinct clusters. The SSR and ISSR markers significantly identified the diversity among the amaranth genotypes, with higher gene diversity values averaging 0.579 and 0.711 for SSR and ISSR, respectively. Heterozygosity average values of 0.723 and 0.601 and the polymorphism information content (PIC) average values of 0.512 and 0.665 for SSR and ISSR, respectively, were observed. The number of primary branches positively correlated with the total number of leaves (r = 0.574**) and marketable leaves (r = 0.591**). Eleven promising trait-specific genotypes were identified with respect to leaf yield, primary branches, and delayed flowering. This research revealed broad phenotypic and genotypic diversity among the amaranth genotypes and identified promising genotypes that can be exploited for the genetic improvement of *Amaranthus* spp. germplasm in Ghana. These findings provide valuable insights into the genetic and phenotypic diversity of Amaranthus, facilitating the selection of high-performing genotypes to harness the contribution of *Amaranthus* spp. in addressing food and nutrition security challenges.

## Introduction

Amaranth *Amaranthus* spp. belongs to the genus *Amaranthus*, a member of the Amaranthaceae family and the order Caryophyllales has more than 60 species [1,2]. The crop is an important indigenous leafy vegetable (ILV) that has high nutritional and medicinal properties for improving human health, alleviating malnutrition and sustaining economies and food security [3–5]. Its leaves are rich in essential minerals such as Ca, Fe, Zn, Mg and K [6,7]. It is also rich in protein, particularly lysine, and contains high levels of vitamins (A, C, and E). It contains antioxidants, such as flavonoids, carotenoids, and phenolic compounds that scavenge free radicals in the human body [8]. In most developing countries, amaranth is a protein-rich vegetable used as an accompaniment to starchy diets in most rural communities [9]. It has gained significance as an important African leafy vegetable and is well known throughout the continent. There is a rising demand for traditional African vegetables, especially amaranth, among urban dwellers [10]. Considering the nutritional attributes and health benefits of amaranth, it plays a role in supplementing dietary needs and can make a significant contribution to the flagship school feeding program of the Government of Ghana. Amaranth is commonly called “aleefu” in Ghana and is mostly grown in the northern part of the country [11]. Young tender leaves and stems are the economically valuable parts of the crop. Amaranth is readily available, less expensive, easy to manage, and is used in the preparation of several forms of food, including vegetable salads, stews, and soups [8].

Several studies have indicated that amaranth thrives well under a wide range of soil conditions and in diverse cropping systems [6,12,13]. This ability makes the crop versatile and easy to establish in diverse growing locations. Its cultivation requires low inputs such as fertilizer, irrigation, and labour. It is a short-lived annual crop that can be harvested within 21 days after planting [6,13]. This attribute can be important for the plant to avoid damage from insect pests and other diseases. This is important for safeguarding natural resources for the benefit of present and future generations, while maintaining ecological balance. Therefore Amaranth can be considered an important crop for the promotion of food and nutrition security [14], as well as a potential source of livelihood improvement for farmers [15]. Despite its remarkable qualities and usefulness, few or no systematic breeding programs and agronomic studies exist to support or promote its cultivation [3], making amaranth an underutilized crop.

As the world’s population continues to grow, the demand for affordable, high-quality food is increasing. However, erratic weather conditions caused by climate change pose serious threats to the survival and biodiversity of amaranth [16], thereby threatening food security and livelihood sustainability. Amaranth is also threatened by changes in agricultural land use due to climate change, urbanization, industrialization, and the introduction of new agricultural technologies including exotic crops. Consequently, the Plant Genetic Resource Research Institute of the Council for Scientific and Industrial Research (CSIR-PGRRI) embarked on a nationwide germplasm collection of amaranths in 2020 and 2021 to preserve its genetic diversity or genetic stock, as well as to promote its future utilization in breeding and research programs. Based on preliminary screening studies by CSIR-PGRRI, promising amaranth accessions were identified that require further evaluation.

Despite its potential and increasing nutritional demand, research on amaranth is generally limited [10,13,15]. Nevertheless, the success of breeding and deployment of promising amaranth varieties largely depends on the availability of a broad diversity of germplasms and the identification of useful traits [16,17]. The availability of such information can also contribute to the effective management of amaranth germplasm at the CSIR-PGRRI gene bank and beyond. Additionally, this study provides useful information for future research and breeding programs. This study will identify promising germplasm that could eventually be improved and released for cultivation, thereby directly contributing to the achievement of at least three important Sustainable Development Goals (SDGs) 1, 2, and 3 of “No poverty, Zero hunger, and Good health and well-being” [18]. The current study was conducted to analyse genetic diversity among twenty-one amaranth genotypes using morphological traits and molecular markers and to screen amaranth genotypes for pest and disease incidence under field conditions.

## Materials and methods

### Experimental site

The experiments were conducted at the University of Ghana’s School Farm in Legon (5.6506^o^ N, 0.1562^o^ W) and the Council for Scientific and Industrial Research-Plant Genetic Resources Research Institute (CSIR-PGRRI), Bunso (6.2871^o^ N, 0.4671^o^ W) in the Coastal Savanna and Forest agro-ecological zones of Ghana, respectively from June, 2023 to September, 2023 at both locations. Access to the study site in Bunso was granted through established partnerships between the University of Ghana and CSIR-PGRRI without requiring permits.

The mean temperature and relative humidity of the two locations during the experiment were monitored using an Easylog USB data logger (EL-USB-2, LASCAR electronics) while rainfall data for Bunso was obtained from the Bunso sub-station of the Ghana Meteorological Agency. Data for Legon was obtained from the Institute of Applied Science and Technology (IAST), University of Ghana, Legon (Table 1). Twenty-one [21] amaranth genotypes e were obtained from the gene banks of CSIR-PGRRI (17 accessions), the World Vegetable Center (AVRDC) (2 improved lines) and farmers’ fields (2 genotypes) (Table 2).

**Table 1:** Climatic conditions at the experimental sites.

Months	Rainfall (mm)		Temperature (^o^C)		Relative humidity (%)	
	Legon	Bunso	Legon	Bunso	Legon	Bunso
Jun −2023	63.80	193.00	26.88	26.55	82.58	84.11
Jul −2023	49.00	336.00	26.86	27.10	85.38	84.78
Aug- 2023	82.80	182.10	26.40	26.16	85.72	87.96
Sep-2023	123.20	191.20	27.43	26.44	83.89	89.40

**Table 2:** List of amaranth genotypes used for the study.

No.	Genotype	Source	No.	Genotype	Source
1	GH10317	CSIR-PGRRI, Bunso	12	GH10186	CSIR-PGRRI, Bunso
2	GH10297	CSIR-PGRRI, Bunso	13	GH10284	CSIR-PGRRI, Bunso
3	GH10295	CSIR-PGRRI, Bunso	14	GH10182	CSIR-PGRRI, Bunso
4	GH10291	CSIR-PGRRI, Bunso	15	GH10141	CSIR-PGRRI, Bunso
5	GH10285	CSIR-PGRRI, Bunso	16	GH10139	CSIR-PGRRI, Bunso
6	GH10282	CSIR-PGRRI, Bunso	17	GH10090	CSIR-PGRRI, Bunso
7	GH10281	CSIR-PGRRI, Bunso	18	OP2023	Farmer’s seed (Opeibea), Accra
8	GH10251	CSIR-PGRRI, Bunso	19	HA2023	Farmer’s seed (Haatso), Accra
9	GH10199	CSIR-PGRRI, Bunso	20	A2004	World Veg. (AVRDC)
10	GH10197	CSIR-PGRRI, Bunso	21	A2002	World Veg. (AVRDC)
11	GH10193	CSIR-PGRRI, Bunso			

### Soil physico-chemical properties at Bunso and Legon

The soil at Legon is classified as Adentan series (Vetic Lixisols) whereas that of Bunso is the Birim series (Dystric Fluvisol). Prior to planting, soil samples were randomly collected from both locations using a soil auger at a depth of 0–15 cm and 15–30 cm for physical and chemical analysis. Soil samples for each depth were then bulked to obtain a composite sample, representative samples were taken and air-dried for the analysis. The soil physical and chemical analysis was performed at the Soil Science Laboratory, Department of Soil Science, University of Ghana. A detailed description of the characteristics of soils at both fields is presented in (Table 3).

**Table 3:** Soil physico-chemical properties at Bunso and Legon.

Chemical properties	Soil properties (0–15 cm)		Soil properties (15–30 cm)	
	Legon	Bunso	Legon	Bunso
pH	5.47	6.6	5.30	6.5
EC (S/m)	0.09	0.13	0.08	0.12
Organic Matter (%)	0.87	3.07	0.32	0.96
Total Nitrogen (%)	0.14	0.17	0.11	0.14
Available P (mg/kg)	9.45	12.56	6.99	7.63
Exchangeable K (cmol/kg)	0.47	0.18	0.26	0.10
Exchangeable Zn (mg/kg)	0.02	0.02	0.01	0.01
Exchangeable Ca (cmol/kg)	1.96	0.99	1.13	1.03
**Physical properties**				
Sand (%)	51.10	16.80	51.60	17.80
Silt (%)	37.00	41.50	11.50	39.20
Clay (%)	11.60	41.70	35.90	43.00
Textural class	Sandy clay loam	Clay loam	Sandy clay loam	Clay loam

EC = Electrical conductivity.

### Field layout and transplanting

At each of the experimental sites, a land size of 32 m x 25 m was ploughed, harrowed, and demarcated into 1.2 m x 4 m experimental units. The experiment was laid out in a Randomized Complete Block Design (RCBD) and replicated three times. Seeds were sown in plastic trays; 30- and 35-day-old seedlings were transplanted onto the field at Bunso and Legon, respectively. There were three rows of eight plants per row, with one plant per stand. A planting distance of 0.6 m x 0.5 m was maintained between and within rows.

### Data collection

#### Agro-morphological characterization.

Twenty-one qualitative and 15 quantitative traits of amaranth plants were characterized at the 50% flowering stage. Qualitative traits such as the presence of leaf axil spines, branching index, prominence of leaf vein, leaf pigment, petiole pigment, leaf shape, inflorescence attitude, inflorescence density index, presence of axillary inflorescence, seed colour and seed coat type were assessed using the AVRDC descriptor for amaranths as a guide [19]. Plant height was measured from the base of the plant at ground level to the topmost tip of the inflorescence with the aid of a meter rule. A digital caliper was used to determine the stem girth at approximately 10 cm above ground, the number of primary branches and leaves per plant were counted and recorded, and leaf size was determined using a leaf area meter (CI-202 LASER AREA METER). The total number of leaves per plant was counted and carefully inspected. Leaves showing signs of damage, yellowing, wilting or diseased were classified as unmarketable. The number of marketable leaves were determined by subtracting the number of unmarketable leaves from the total count. The chlorophyll content of the leaves was measured on six new and fully developed leaves from three plants per experimental unit using non-destructive method. A SPAD chlorophyll meter, (KONICA MINOLTA) was used.

### Molecular characterization

#### Genomic DNA extraction.

A modified cetyltrimethylammonium bromide (CTAB) method [20] was used for DNA extraction. Approximately 50 mg of young and fresh tender leaves of amaranth samples were weighed into 1.5 ml micro-centrifuge tubes and macerated with sterilized plastic pestles to a fine paste. A 1 mL extraction buffer (7% CTAB, 1% polyvinylpyrrolidone, 0.1 m Tris–HCl, 1.4 m NaCl, 20 mm EDTA pH 8.0), 300 µL 20% sodium dodecyl sulphate and 0.5% mercaptoethanol (v/v) were then, added and mixed by vortexing. The samples were incubated at 55 °C for 15 min and the homogenates were extracted with phenol/chloroform/isoamyl alcohol (25:24:1, v/v/v). The aqueous phase was separated by centrifugation and incubated with RNase A (10 μg/ml) at 37 °C for 20 min. After transferring the upper aqueous phase into new micro-centrifuge tubes, DNA was precipitated by adding 0.7 volume cold isopropanol and incubating on ice for 15 min. After centrifugation (10 min at 10 000 revs/min) to collect DNA, the nucleic acid was further purified and re-precipitated by adding 250 µL ice-cold 70% ethanol and 50 µL of 7.5 M ammonium acetate (pH 7.7) and centrifuging at 10 000 revs/min for 5 min. The final pellet was washed twice in 500 µL of ice-cold 70% ethanol, air-dried and re-suspended in 100 µL Tris-EDTA (TE) (10 mM Tris, 1 mM EDTA) buffer [pH 8.0] and stored at −20 °C for further studies.

#### Polymorphic chain reaction.

A set of simple sequence repeat (SSR) and inter-simple sequence repeat (ISSR) primers (Table 6) were used for the polymorphic chain reaction (PCR) amplification. For each of the 21 samples, PCR amplification of the extracted DNA was performed using BIO-RAD PTC-220 thermocycler (Dyad MJ Research) in a total volume of 25 µl containing 12.50 µl of OneTaq Quick-Load 2x Master Mix with Standard Buffer, 10 µM each of the primer, ~ 5 ng of genomic DNA. The PCR (T100 Thermal Cycler, Bio-Rad, Hercules, CA, USA) reaction comprised 35 cycles, with strand separation at 94 ^◦^C for 30 s, an annealing time of 30 s at 60 ^◦^C, and an extension time of 1 min at 72 ^◦^C. PCR products were separated by gel electrophoresis on a 1.2% (w/v) agarose gel in 1x TAE buffer (40 mM Tris-acetate, 1 mM EDTA, pH 8.0) at 80 V for 90 min. The gels were stained with Ethidium bromide and visualized with UV light using a Cole Palmer FLUO-LINK FLX (Applied Biosystems, USA) apparatus and photographed for later assessment.

#### Scoring of data.

**Plate 1:**
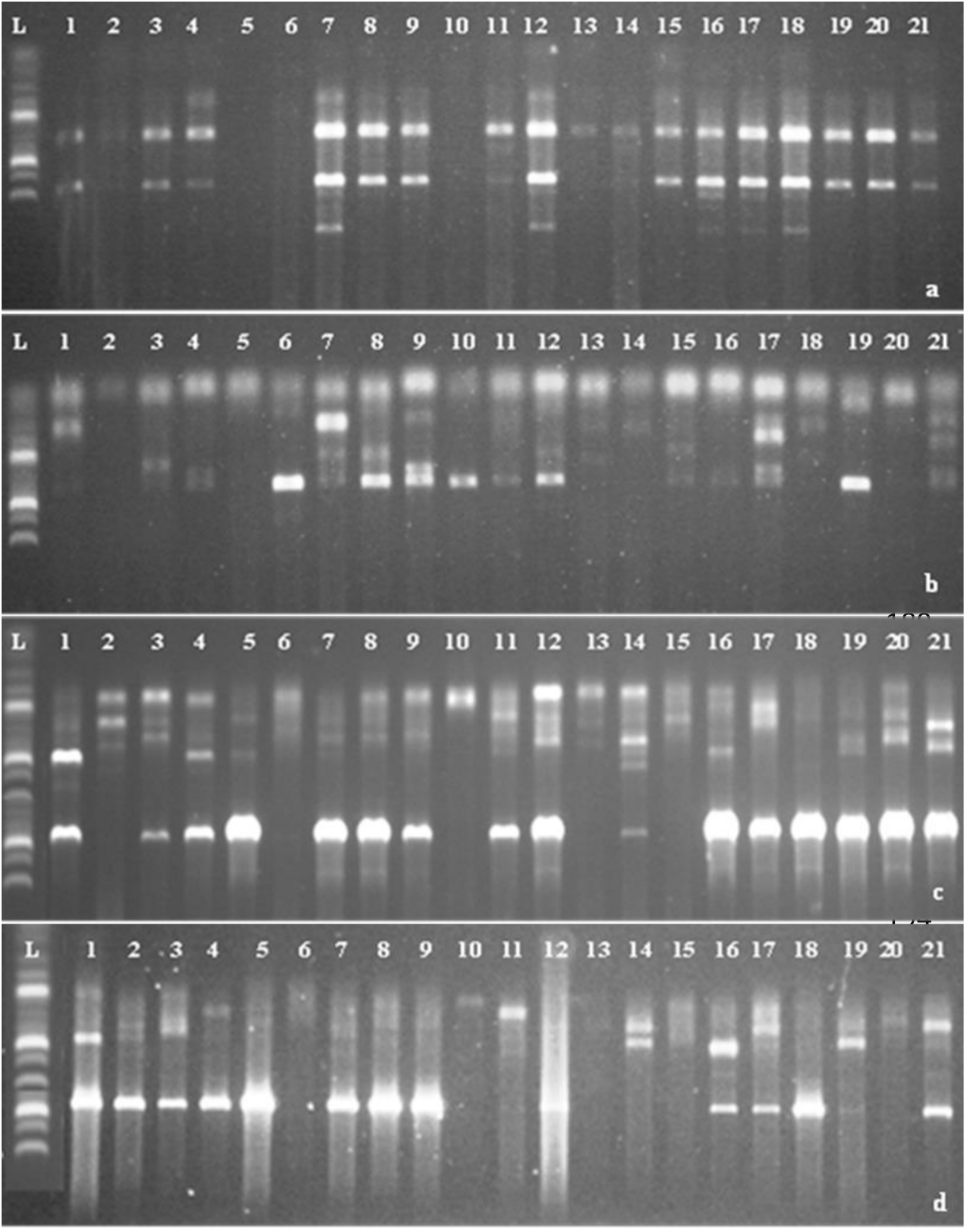
Agarose gel electrophoresis of amplified 21 amaranth genotypes obtained with SSR and ISSR markers. (a) SSR gpsb067-F, SSR gpsb067-R (b) SSR Xisep0107-F, SSR Xisep0107-R (c) ISSR 26 (d) ISSR 21 (L) 100 bp ladder [1] GH10317 [2] GH10297 [3] GH10295 [4] GH10291 [5] GH10285 [6] GH10282 [7] GH10281 [8] GH10251 [9] GH10199 [10] GH10197 [11] GH10193 [13] GH10186 [14] GH10284 [15] GH10182 [16] GH10141 [17] GH10139 [18] GH10090 [19] OP2023 [19] HA2023 [20] A2004 [21] A2002.

Clear and distinct bands amplified by SSR and ISSR primers were scored for the presence (1) or absence (0) of the corresponding band among amaranth genotypes as binary data [16] (Plate 1).

### Assessment of insect pest incidence

**Plate 2:**
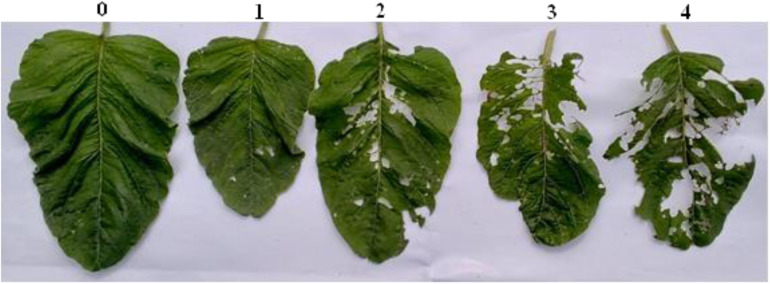
Rating of damaged leaves. 0: no visible damage on the leaves – leaves marketable/high value; **1**: 1–25% of the leaves damaged – Mild damage/few window holes; **2**: 26–50% of the leaves damaged – Moderately damaged/reduced market value; **3**: 51–75% of the leaves damaged -Severe infestation/not marketable and **4**: > 75% leaves damaged – Very severe/devastated.

All amaranth plants in the middle row of each treatment plot were inspected for the presence of and damage caused by insect pests. Damage symptoms, such as ragged leaf margins, skeletonized leaves, window holes in leaves, and rolling of leaves, were recorded (Plate 2). A rating scale was used to grade the levels of damage from (0–4) where, 0 = no damage and 4 = severely damaged/devastated (Plate 2). The presence of whiteflies, leaf hoppers and leaf rollers/skeletonizers was counted per plant and recorded. The data are expressed as percentages of incidence and severity.

### Assessment of disease incidence

All plants in each plot were inspected for disease incidence and symptoms such as wilt, wet rot and stem canker and the scores obtained were recorded. The data collected are expressed as percentages of incidence and severity. Diseased plant samples were collected into a labelled zip lock bag and transported to the plant pathology laboratory of the Department of Crop Science, University of Ghana, Legon. The diseased plants were cultured on a potato dextrose agar (PDA) media. Pest and disease incidence and severity were calculated using the methods of [1,21].


$$\text{Incidence}\left(\%\right)=\frac{\text{number}\;\text{of}\;\text{infected}\;\text{plants}}{\text{total}\;\text{plants}\;\text{inspected}\;}*\text{100}$$

$$\text{Severity}(\%)=\frac{\text{sum}\;\text{of}\;\text{rating}\;\text{of}\;\text{plants}}{\text{total}\;\text{plants}\;\text{inspected}\;*\;\text{maximum}\;\text{grade}\;\text{on}\;\text{scale}\;}*100$$

### Data analyses

The software XLSTAT (2014.5.03) was used for the analysis of variance, and Pearson’s correlation analysis was used to determine the relationships between traits. Microsoft Excel 2013 was used to plot the graphs. PowerMarker statistical software (version 3.1) was used to estimate the gene diversity, heterozygosity and molecular weight of the bands separated on the gel. The polymorphism information content (PIC) value was calculated based on allele frequency using the given formula.


$$\text{Polymorphism}\;(\%)\;=\frac{\text{number}\;\text{of}\;\text{polymorphic}\;\text{bands}}{\text{total}\;\text{number}\;\text{of}\;\text{bands}}*\text{100}$$

## Results

### Analysis of variance of quantitative traits of amaranth genotypes

The results from the combined analysis of variance across the two experimental locations revealed that genotype showed highly significant (P < 0.001) differences for majority of the traits except for unmarketable leaves (UML) and chlorophyll content (CC). Again, apart from unmarketable leaves (UML), leaf length (LL), leaf width (LW) and thousand seed weight (TSW), highly significant differences (P < 0.001) were observed among the experimental locations for most of the traits tested. Similarly, traits such as leaf area (LA), petiole length (PL) and days to 50% flowering were significantly (P < 0.01) affected by the genotype by environment interactions indicating that some traits performed better depending on the environment (Table 4).

**Table 4:** Analysis of variance of the quantitative traits of the 21 amaranth genotypes.

Source of variation	Vegetative traits			Leaf characteristics				
	PH	SG	NPB	TTL	UML	ML	LA	LL
Genotype	37929.2***	1420.7*	811.4*	31536.9*	135.3	29457.2*	76427.8***	174.5*
Location	9519.5***	568.2**	1091.2***	30844.6***	14.3	32211.2***	2893.4**	18.4
Genotype **x** location	14267.2*	1143.3	370.6	23927.2	142.6	22161.6	18412.2***	184.2*
**Continuation**								
**Source of variation**	**Leaf characteristics**			**Reproductive traits**				
	**LW**	**CC**	**PL**	**DF1** ^**ST**^	**DF50%**	**IDF**	**TSW**	
Genotype	535.0***	735.8	283.3***	8146.8***	6005.4***	2429.3***	0.28***	
Location	4.3	2008.8***	16.1*	85.8*	398.2***	114.3*	0.02	
Genotype x location	107.6	681.5	233.9***	300.5	851.4**	937.7*	0.13	

The values in the table represent the sum of squares, * = significant at P < 0.05, ** = significant at P < 0.01, *** = significant at P < 0.0001; **PH **= plant height, **SG** = stem girth, **NPB** = number of primary branches, **TTL **= total number of leaves, **UML** = unmarketable leaves, **ML** = marketable leaves, **LA** = leaf area, **LL **= leaf length, **LW** = leaf width, **CC** = chlorophyll content, **PL** = petiole length, **DF 1**^**ST**^ = Days to first flowering, **DF50%** = days to 50% flowering, **IDF** = intervals between 1^st^ and 50% flowering and **TSW** = thousand seed weight.

### Mean performances of leaf characteristics, branching index and days to 50% flowering of 21 amaranth genotypes

All the tested genotypes had large leaves (length > 10 cm) (Table 5). Based on leaf yield (total leaves), number of primary branches (NPB), days to 50% flowering (DF50%) and marketable leaves (ML), the top eleven genotypes - GH10281, GH10182, GH10285, GH10317, GH10284, GH10291, GH10090, GH10193, GH10186, GH10141 and GH10297 were selected. Among them, the six highest-performing genotypes produced 1.6–27% more leaves (TTL) than the best check (A2004) in Legon with 127 (TTL) (Table 6). These genotypes exhibit a higher number of branches, more leaves, and delayed days to 50% flowering (Tables 5 and 6).

**Table 5:** Mean performances of leaf characteristics, of 21 amaranth genotypes.

Genotypes	Total leaves		Marketable leaves		Leaf length (cm)	
	Bunso	Legon	Bunso	Legon	Bunso	Legon
A2002	71.9 ± 3.0^a^	116.1 ± 16.6^a^	65.3 ± 2.3^a^	109.0 ± 15.9^a^	19.7 ± 0.7^a^	18.8 ± 1.2^a^
A2004	143.3 ± 14.2^a^	127.3 ± 22.9^a^	133.1 ± 1.1^a^	119.8 ± 23.3^a^	22.4 ± 0.5^a^	21.2 ± 2.4^a^
GH10090	88.8 ± 19.8^b^	158.3 ± 17.9^a^	83.0 ± 20. 6^b^	152.9 ± 17.6^a^	21.7 ± 1.9^a^	24.9 ± 1.4^a^
GH10139	94.7 ± 19.7^a^	112.7 ± 2.6^a^	86.9 ± 22.0^a^	109.0 ± 2.3^a^	21.4 ± 0.6^a^	22.3 ± 0.4^a^
GH10141	41.7 ± 8.0^b^	142.5 ± 12.1^a^	38.3 ± 8.2^b^	136.2 ± 11.9^a^	23.0 ± 0.3^a^	24.9 ± 0.2^a^
GH10182	129.3 ± 23.9^a^	116.5 ± 19.8^a^	120.3 ± 23.7^a^	109.3 ± 20.1^a^	24.0 ± 0.9^a^	20.9 ± 1.0^a^
GH10186	70.1 ± 24.7^a^	118.1 ± 5.3^a^	63.0 ± 24.2^b^	110.5 ± 6.2^a^	22.3 ± 0.6^a^	22.7 ± 0.9^a^
GH10193	94.2 ± 24.9^a^	125.3 ± 4.8^a^	86.9 ± 23.5^a^	120.9 ± 5.6^a^	22.2 ± 0.5^a^	22.8 ± 0.3^a^
GH10197	71.7 ± 9.6^a^	79.7 ± 10.6^a^	65.1 ± 7.7^a^	75.9 ± 10.8^a^	21.1 ± 0.5^a^	21.5 ± 0.9^a^
GH10199	78.6 ± 14.3^a^	123.2 ± 34.6^a^	73.6 ± 14.6^a^	116.4 ± 35.0^a^	20.9 ± 0.6^a^	19.1 ± 1.5^a^
GH10251	85.9 ± 14.7^a^	102.0 ± 13.7^a^	80.3 ± 13.8^a^	93.6 ± 12.0^a^	23.5 ± 1.0^a^	20.7 ± 0.9^a^
GH10281	103.0 ± 44.8^b^	161.3 ± 31.5^a^	95.5 ± 41.2^b^	153.3 ± 31.2^a^	25.4 ± 0.3^a^	21.3 ± 1.3^b^
GH10282	72.2 ± 4.0^a^	87.6 ± 1.9^a^	64.1 ± 1.2^a^	84.1 ± 2.7^a^	19.4 ± 0.3^a^	21.7 ± 1.4^a^
GH10284	88.6 ± 30.8^b^	155.0 ± 8.3^a^	81.6 ± 30.7^b^	147.2 ± 7.8^a^	20.7 ± 0.4^a^	23.1 ± 0.9^a^
GH10285	103.2 ± 15.4^a^	129.8 ± 12.8^a^	95.9 ± 15.5^a^	124.5 ± 12.7^a^	25.1 ± 1.3^a^	21.5 ± 0.7^a^
GH10291	99.3 ± 11.0^a^	133.2 ± 8.1^a^	93.9 ± 10.5^a^	127.2 ± 7.9^a^	23.4 ± 1.4^a^	21.0 ± 0.2^a^
GH10295	98.9 ± 17.3^a^	127.2 ± 19.9^a^	93.0 ± 16.6^a^	122.4 ± 21.0^a^	21.8 ± 0.8^a^	22.1 ± 1.7^a^
GH10297	84.5 ± 5.4^a^	109.7 ± 4.7^a^	76.6 ± 3.9^a^	105.2 ± 4.2^a^	21.3 ± 1.7^a^	21.3 ± 1.1^a^
GH10317	101.9 ± 6.0^a^	143.6 ± 36.1^a^	94.9 ± 7.4^a^	136.1 ± 35.8^a^	21.7 ± 0.6^a^	22.6 ± 3.2^a^
HA2023	108.3 ± 12.4^a^	105.5 ± 13.7^a^	103.2 ± 12.7^a^	99.3 ± 12.8^a^	23.3 ± 0.4^a^	20.1 ± 1.2^a^
OP2023	96.0 ± 8.1^a^	108.5 ± 19.1^a^	88.5 ± 5.8^a^	101.7 ± 17.6^a^	27.3 ± 3.6^a^	21.1 ± 1.0^b^
**LSD**	**10.56**		**10.22**		**0.77**	

Means in a row for a particular variable followed by the same alphabets are not significantly different at p = 0.05. Mean** **±** **standard error.

**Table 6:** Mean performances of branching index and days to 50% flowering of 21 amaranth genotypes.

Genotypes	Number of primary branches			DF50% (days)	
	Bunso	Legon		Bunso	Legon
A2002	10.1 ± 0.9^a^	15.1 ± 1.4^a^		59.3 ± 1.5^a^	60.7 ± 0.3^a^
A2004	13.7 ± 0.4^a^	16.1 ± 0.9^a^		53.7 ± 2.7^a^	59.0 ± 1.0^a^
GH10090	10.9 ± 1.6^b^	18.7 ± 2.3^a^		41.0 ± 1.7^a^	35.3 ± 1.5^a^
GH10139	10.9 ± 1.8^a^	13.7 ± 0.8^a^		44.3 ± 3.3^a^	42.3 ± 1.3^a^
GH10141	8.4 ± 1.7^b^	16.7 ± 1.0^a^		45.0 ± 1.0^a^	41.0 ± 0.0^a^
GH10182	12.9 ± 1.5^a^	15.2 ± 1.2^a^		45.3 ± 3.0^a^	48.7 ± 1.9^a^
GH10186	8.7 ± 2.1^a^	15.1 ± 0.9^a^		51.3 ± 0.3^a^	44.3 ± 1.3^b^
GH10193	12.9 ± 0.6^a^	15.6 ± 1.9^a^		48.0 ± 2.3^a^	49.3 ± 0.3^a^
GH10197	8.8 ± 0.4^a^	12.5 ± 0.7^a^		34.0 ± 0.0^a^	30.0 ± 2.5^b^
GH10199	11.1 ± 1.0^a^	16.8 ± 2.3^a^		39.7 ± 1.9^a^	35.7 ± 2.3^a^
GH10251	8.7 ± 1.4^a^	14.6 ± 0.7^a^		41.0 ± 1.7^a^	32.3 ± 3.0^b^
GH10281	13.2 ± 0.8^a^	16.6 ± 1.6^a^		45.3 ± 3.0^a^	45.0 ± 0.0^a^
GH10282	10.5 ± 0.1^a^	13.6 ± 0.8^a^		36.3 ± 2.3^a^	26.0 ± 1.5^b^
GH10284	10.4 ± 2.0^b^	18.0 ± 0.6^a^		46. ± 1.3^b^	34.3 ± 3.3^a^
GH10285	13.0 ± 1.1^b^	29.3 ± 11.3^a^		43.0 ± 4.0^a^	41.0 ± 0.0^a^
GH10291	14.3 ± 1.7^b^	27.5 ± 9.7^a^		38.3 ± 1.7^a^	36.7 ± 3.0^a^
GH10295	10.4 ± 0.8^a^	16.9 ± 1.6^a^		42.0 ± 1.0^a^	43.7 ± 2.7^a^
GH10297	10.8 ± 1.3^a^	14.7 ± 1.2^a^		45.7 ± 3.0^a^	45.3 ± 2.6^a^
GH10317	11.8 ± 0.1^a^	18.5 ± 1.7^a^		41.0 ± 2.0^a^	40.0 ± 1.0^a^
HA2023	12.7 ± 0.9^a^	16.5 ± 1.2^a^		40.0 ± 1.0^a^	30.7 ± 1.5^b^
OP2023	10.7 ± 1.1^a^	16.9 ± 1.2^a^		50.0 ± 1.0^a^	35.0 ± 5.0^b^
**LSD**	**1.60**		**1.32**		

Means in a row for a particular variable followed by the same alphabets are not significantly different at p = 0.05. Mean** **±** s**tandard error.

### Frequency distribution of qualitative traits of amaranth genotypes

Most of the characteristics studied showed significant variability among the amaranth genotypes (Table 7).

**Table 7:** Minimum and maximum values for all the 15 quantitative traits.

Trait	Minimum value	Maximum value
Chlorophyll content	30.54	96.56
Unmarketable leaves	1.80	17.20
Leaf area	63.90	240.60
Leaf length	16.20	34.40
Leaf width	6.82	35.21
Number of branches	6.00	51.00
Marketable leaves	26.40	196.60
Petiole length	11.00	26.00
Plant height	125.00	240.00
Stem girth	15.66	76.22
Total leaves	31.80	202.00
Days to first flowering	10.00	58.00
Days to 50% flowering	23.00	62.00
Thousand seed weight	0.27	0.95
Interval between flowering	0.00	35.00

The amaranth genotypes studied had many branches, however, the distribution of branches on the stem differed. Approximately 66.7% had branches near the base of the stem whereas 33.3% had branches all along the stem. Regarding stem pigmentation, 76.2% of the genotypes presented a green pigment, 9.5% presented a mixture of green/pink another 9.5% presented a light green pigment and 4.8% presented a yellowish green pigment. The absence of spines in the leaf axil dominated (90.5%). Over 90% of the genotypes presented normal green leaf pigmentation. Lanceolate and elliptical were the dominant leaf shapes contributing 52.4% and 38.1% respectively to the variation in leaf shape. Approximately14.3% showed rugose prominence of leaf the vein and the rest were smooth. The majority of the genotypes (85.7%) presented a green petiole colour, approximately 90% did not have flowers at the leaf axile, and only 9.5% presented this trait. A wide variation in the flower density index was observed. Approximately 61.9% had an intermediate density index while 28.6% and 9.5% had lax and dense density indices, respectively. Green flowers were predominant in 90.5% of the genotypes, and 52.4% of the seeds were black, and 47.6% were brown (Table 8). In contrast, all the remaining traits were monomorphic among the genotypes.

**Table 8:** Descriptors and frequency distributions of 11 qualitative amaranth traits.

Traits	Descriptors (Code)	Frequency (%)
Branching index	Many (all near the base of the stem) (3) Many (all along the stem) (4)	66.7 33.3
Stem pigment	Green (1) Green/pink mixture (x) Light green (5) Yellowish green (6)	76.2 9.5 9.5 4.8
Spines in leaf axils	Absent (1) Mixture (3)	90.5 9.5
Leaf pigmentation	Normal green (8) Dark green (9)	90.5 9.5
Leaf shape	Lanceolate (1) Elliptical (2) Deltoid (8)	52.4 38.1 9.5
Prominence of leaf veins	Smooth (1) Rugose (veins prominent) (2)	85.7 14.3
Petiole pigmentation	Green (1) White (5) green/pink (6)	85.7 4.8 9.5
Presence of axillary inflorescence	Absent (1) Present (2)	90.5 9.5
Inflorescence density index	Lax (3) Intermediate (5) Dense (7)	28.6 61.9 9.5
Inflorescence colour	Green (2) Dark green (5)	90.5 9.5
Seed colour	Brown (4) Black (5)	47.6 52.4

### Cluster analysis based on quantitative, qualitative and molecular traits

The dendrogram based on the quantitative, qualitative and molecular traits, grouped all genotypes into three major clusters: I, II, and III (S1–S3 Figs). Cluster I, which was based on the quantitative traits of amaranth, had eight genotypes including the two improved check entries A2004 and A2002. The members of this group produced more primary branches, more total leaves, and the most delayed number of days to 50% flowering (S1 Fig). Cluster II had 11 genotypes including the two local check entries OP2023 and HA2023. The genotypes in this group presented distinct similarities in terms of leaf length, high chlorophyll content and the least 1000 seed weight. However, cluster III had two genotypes GH10282 and GH10197 whose plant height, leaf area and number of days to 50% flowering were similar. Based on the qualitative properties, three cluster groups were also identified (S2 Fig). Cluster I included nine genotypes with lanceolate leaves. Cluster II included 10 genotypes which had elliptical leaf shape and brown seed colour whereas, cluster III had two genotypes GH10282 and GH10297, which had rugose leaf lamina prominence, a dark green leaf colour, a deltoid leaf shape and the presence of axillary flowers with dark green flower colour. The dendrogram developed using the binary data generated from the extracted DNA of all 21 amaranth genotypes clustered all the genotypes into three distinct clusters (I, II and III) (S3 Fig). Each of the clusters was again divided into two or more sub-clusters. Cluster I consisted of nine genotypes all from the CSIR-PGRRI seed gene bank, cluster II contained 10 genotypes including the two improved lines from the World Vegetable Center and one of the local checks. Cluster III had the least number of genotypes, comprising three genotypes, namely, GH10193, GH10199 and HA2023 (local check).

### Cluster variable analysis

Cluster variable analysis was performed to group the variables into clusters that share common characteristics. The analysis grouped the tested variables into six clusters (Table 9). The first three clusters (I, II, and III) consisted of three quantitative traits each, whereas the last three clusters (IV, V, and VI) consisted of two traits each. Marketable leaves contributed more than 80% of the variation in cluster I. Similarly, cluster I had the highest (0.164)total proportion of variation. Plant height, days to 50% flowering, chlorophyll content, petiole length and leaf area were the representative variables of the clusters (II, III, IV, V and VI) and the cluster proportion of variations of 0.61, 0.72, 0.78, 0.54 and 0.97 respectively (Table 9).

**Table 9:** Cluster variable analysis of amaranth genotypes.

Cluster	Number of Members	Most Representative Variable	Cluster Proportion of Variation Explained	Total Proportion of Variation Explained
I	3	Marketable leaves	0.821	0.164
II	3	Plant height	0.614	0.123
III	3	Days to 50% flowering	0.72	0.144
IV	2	Chlorophyll content	0.775	0.103
V	2	Petiole length	0.541	0.072
VI	2	Leaf area	0.97	0.129

### Correlation coefficient of 15 quantitative variables measured on 21 amaranth genotypes

The combined analysis revealed significant (P < 0.05) differences as well as positive and negative correlations (*r*) among the quantitative traits (Table 10). Plant height was positively and strongly correlated with stem girth (r = 0.731***). Similarly, the number of primary branches had positive correlation with total leaves (r = 0.574**) and marketable leaves (r = 0.591**). Also, total number of leaves, number of unmarketable leaves, and marketable leaves, positively correlated r = 0.535** and r = 0.998*** respectively. In contrast, total number of leaves negatively correlated with leaf area (r = −0.505**) and leaf width (r = −0.598***). Positive correlations of r = 0.487* and r = 0.520* were observed between number of unmarketable leaves, marketable leaves, and days to 50% flowering, respectively. Inversely, number of marketable leaves had high negative correlation with leaf area r = −0.500* and leaf width r = −0.595**. A strong positive correlation (r = 0.940***) was noted between leaf area and leaf width whereas, leaf area and days to 50% flowering correlated negatively (r = −0.556**). The correlation between chlorophyll content and interval between first and 50% flowering was positively strong (r = 0.550**). Again, days to first flowering showed a highly positive correlation with days to 50% flowering (r = 0.838***).

**Table 10:** Correlation coefficient of 15 quantitative variables measured on 21 amaranth genotypes.

	PH	SG	NPB	TTL	UML	ML	LA	LL	LW	CC	PL	DF1^ST^	DF50%	IDF
**SG**	0.731***													
**NPB**	−0.060	0.140												
**TTL**	−0.080	0.025	0.574**											
**UML**	0.102	0.155	0.054	0.535**										
**ML**	−0.091	0.015	0.591**	0.998***	0.487*									
**LA**	0.478*	0.465*	−0.285	−0.505**	−0.335	−0.500*								
**LL**	−0.239	−0.109	0.275	0.353	0.078	0.361	0.115							
**LW**	0.485*	0.470*	−0.355	−0.598***	−0.352	−0.595**	0.940***	−0.107						
**CC**	−0.183	−0.206	−0.006	−0.099	−0.218	−0.087	−0.029	−0.067	−0.015					
**PL**	0.157	0.024	−0.300	−0.232	0.195	−0.251	0.300	0.081	0.144	0.204				
**DF1ST**	0.444*	0.232	−0.097	0.197	0.342	0.181	−0.352	−0.334	−0.302	−0.156	−0.037			
**DF50%**	0.264	0.009	−0.011	0.336	0.520*	0.313	−0.574	−0.066	−0.556**	−0.275	−0.010	0.838***		
**IDF**	−0.286	−0.522*	−0.262	−0.281	−0.239	−0.275	−0.236	−0.448*	−0.104	0.550**	−0.079	−0.116	−0.162	
**TSW**	0.222	0.210	−0.259	0.052	0.400	0.027	0.145	0.068	0.117	−0.051	0.355	0.207	0.198	−0.072

* = Significant at P < 0.05, ** = significant P < 0.01, *** = significant at P < 0.0001; **PH** = plant height, **SG** = stem girth, **NPB** = number of primary branches, TTL = total number of leaves, UML = unmarketable leaves, ML = marketable leaves, **LA** = leaf area, **LL** = leaf length, **LW** = leaf width, **CC** = chlorophyll content, **PL** = petiole length, **DF 1**^**ST**^ = Days to first flowering, **DF50%** = days to 50% flowering, **IDF** **= **intervals between 1^st^ and 50% flowering and **TSW** = thousand seed weight.

### Molecular diversity analysis

The genetic relatedness among the 21 amaranth genotypes was estimated using six SSR and four ISSR markers. Gene diversity based on the SSR markers ranged from 0.432 to 0.711, with an average of 0.579, while that of the ISSR marker ranged from 0.647 to 0.805, with an average of 0.711 by the ISSR markers. Higher values of Heterozygosity were observed within the SSR markers as compared to the ISSR markers. Heterozygosity based on the SSR markers ranged from 0.550 (Xtxp321) to 0.882 (gpsb067) averaging 0.723 while values based on the ISSR makers ranged between 0.400 (ISSR22) and 0.789 (ISSR25) averaging 0.601. Polymorphic information content (PIC) values for SSR ranged from 0.339 (Xtxp273) to 0.665 (Xtxp321) with an average of 0.512 per primer, whereas PIC values based on the ISSR markers were in the range of 0.591 (ISSR26) and 0.777 (ISSR 22) with an average of 0.665 per primer (Table 11).

**Table 11:** Resolving power analysis of the ISSR and SSR primers.

Primer name	Sequence (5’ → 3’)	Tm	TN	GD	He	PIC
**ISSR primers**						
ISSR 21	GCCTCTCTCTCTCTCTCT	56.0	18	0.70	0.71	0.65
ISSR 22	GACTCTCTCTCTCTCTCT	53.7	18	0.81	0.40	0.78
ISSR 25	GCCCTCTCTCTCTCTCTCT	58.8	19	0.70	0.79	0.64
ISSR 26	GCACTCTCTCTCTCTCTCT	56.7	19	0.65	0.50	0.59
**SSR primers**						
gpsb067-F	TAGTCCATACACCTTTCA	51–63	18	0.54	0.88	0.44
gpsb067-R	TCTCTCACACACATTCTTC	51–63	19			
gpsbo089-F	ATCAGGTACAGCAGGTAGG	51–63	19	0.53	0.81	0.45
gpsb089-R	ATGCATCATGGCTGGT	51–63	16			
Xisep0107-F	GCCGTAACAGAGAAGGATGG	51–63	20	0.57	0.76	0.54
Xisep0107-R	TTTCCGCTACCTCAAAAACC	51–63	20			
Xcup11-F	TACCGCCATGTCATCATCAG	51–63	20	0.69	0.70	0.63
Xcup11-R	CGTATCGCAAGCTGTGTTTG	51–63	20			
Xtxp273-F	GTACCCATTTAAATTGTTTGCAGTAG	51–63	26	0.43	0.63	0.34
Xtxp273-R	CAGAGGAGGAGGAAGAGAAGG	51–63	21			
Xtxp321-F	TAACCCAAGCCTGAGCATAAGA	51–63	22	0.71	0.55	0.67
Xtxp321-R	CCCATTCACACATGAGACGAG	51–63	21			

**Tm** = temperature, **TN** = total nucleotides, **GD** = gene diversity, **He** = Heterozygosity, **PIC** = polymorphism information content.

### Assessment of insect pest damage and disease incidence on amaranth

The incidence of wet rot and stem canker diseases were common among the genotypes. However, wet rot was higher, over 70% compared to stem canker (S4 Fig). Whiteflies were the most dominant insect pests followed by leaf rollers/skeletonizers (S5 Fig). Generally, pest and disease incidence was higher under the Bunso condition as compared to that of Legon (S6 and S7 Figs). The genotype GH10141 had the highest percentage disease incidence 70.9% and severity index 56.9% at Bunso. Genotypes GH10182, GH10193, GH10199, GH10295, GH10284 and GH10285 showed minimum disease symptoms, below 10% (S6 Fig). Pests’ incidence followed the same pattern as the disease incidence with high incidence at Bunso compared to Legon (S7 Fig). The leaves were the main target parts for the insects compared with the other vegetative parts. All amaranth genotypes showed some level of insect damage.

### Identification of promising genotypes with desirable traits

Amaranth genotypes were ranked in a descending order and the best performing genotypes in each location with high mean value of desirable yield traits (Tables 5 and 6) were selected as promising. The selected genotypes are listed in Table 12. Only GH10281 and GH10284 performed best in all the four yield attributing traits. Again, these genotypes showed low pest and disease incidence.

**Table 12:** Identification of promising genotypes with desirable traits.

S/N	Desirable traits	Genotypes
1	Total leaves	GH10281, GH10090, GH10284, GH10317, GH10141, GH10291, GH10285, GH10182
2	Marketable leaves	GH10281, GH10090, GH10284, GH10141, GH10317, GH10291, GH10285
3	Number of primary branches	GH10285, GH10291, GH10090, GH10317, GH10284, GH10141, GH10281
4	Days to 50% flowering	GH10186, GH10193, GH10182, GH10284, GH10297, GH10281

## Discussion

### Diversity based on agro-morphological and molecular assessment of amaranth

The objective of this study was to analyze the genetic diversity among 21 amaranth genotypes using agro-morphological traits, and molecular markers, and to screen amaranth genotypes for pest and disease incidence under field conditions. This serves as an initial step to identifying promising genotypes in terms of leaf yield attributes that can be directly utilized or included in amaranth improvement programs. Many of the amaranth genotypes exhibited a wide range of phenotypic variations, which makes it more likely to find promising germplasm for the genetic improvement of desirable attributes for leaf yield. This is evidenced by the wide significant differences observed for most of the traits, including plant height (PH), stem girth (SG), number of primary branches (NPB), total number of leaves (TTL), marketable leaves (ML), days to 50% flowering (DF50%), branching index (BI), leaf pigmentation (LPG), leaf shape (LS), flower colour (FC) and seed colour (SC) tested in this study. Several factors, such as the origins of the genotypes collected and inherent variability present in amaranth genotypes may be responsible for the observed variations in the expression of traits. Shukla et al. [22] reported a firm interrelationship between diversity among genotypes and the geographical origin of vegetable amaranth. This is consistent with the observations of [4,23] who reported higher levels of phenotypic diversity among amaranth genotypes. Nyasulu et al. [24] attributed morphological variations in amaranth accessions that were evaluated in the same environment to genotypic distinctiveness among the accessions. Earlier studies have reported several dominant genes that control patterns of pigmentation, such as the presence of betacyanin determines the expression of wide range of pigments among amaranth species [24]. It is undoubtedly clear that the tested genotypes in this study are composed of several important genes that expressed wider variations in terms of stem, leaf, petiole, inflorescence, and seed colour. The strong relationship between location and agronomic variables such as plant height, number of primary branches, total leaves, marketable leaves, and days to 50% flowering indicate that the environment has an impact on the expression of traits. The resulting variation present among genotypes is a quality attribute of a good genetic material that can directly be included in the development of improved varieties [25]. Again, the varied performance of amaranth genotypes presents a great opportunity to identify and select superior genotypes for specific environmental conditions. The understanding of genetic differences of individual traits seems not enough, however, knowledge on genetic diversity that comprises several traits at the same time is very key in selection for plant breeding. Estimation of genetic diversity using agro-morphological markers is easy to perform despite often being influenced by environmental factors [26]. Meanwhile, morphological markers allow for estimation of diversity among plant species under varied environments. In the current research, green leaf pigment was found to be dominant among the genotypes tested. Leaf shape, size and colour of amaranth are quality attributes that attract the interest of producers and consumers [13,26]. Thus, it has significant socio-economic influence on consumer preference and natural selection as it determines the predominant traits in a particular community. Amaranth varieties that have green leaves are mostly preferred in diets on the African continent compared to varieties with red leaves which are preferred by the Chinese [4,23,24].

The average PIC values of 0.665 and 0.512 for all the four ISSR and six SSR markers respectively indicated high allelic diversity among the amaranth genotypes and informativeness of the markers. Serrote et al. [27] opined that markers that have a PIC value ranging between 0.5–1.0 are highly informative for genetic studies as they express significant variability among alleles allowing for better discrimination between individual plant species or populations. The four ISSR and six SSR markers used in this study separately detected high levels of variability in the tested genotypes. This could mean that ISSR and SSR markers have high discriminatory power [28] combined with greater number of alleles evenly distributed at the locus gives an indication of genetic diversity in plant species. The characteristic high PIC values of the markers used in this study implies they have greater potential to be used in genetic diversity analysis in amaranth [16].

Additionally, the higher values of gene diversity and Heterozygosity detected among the studied genotypes using ISSR and SSR markers may imply that, the markers capture genetic variation or the utility in distinguishing among the individuals to reveal their information content for genetic analysis was not limited. The finding in this research confirms the report by [27]. The authors stated that a marker’s heterozygosity, which is based on the quantity and frequency of alleles in the population, is the probability that a species will be heterozygous at the marker location. The range of values for heterozygosity is between 0 and 1, zero indicates no heterozygosity while 1 indicates several alleles with equal frequency. The diversity in alleles plays an essential role in the survival of plant species and enables them adapt to diverse environmental conditions [29,30].

Plant height, number of primary branches and marketable leaves for instance were better at Legon than at Bunso. However, amaranth genotypes had less chlorophyll content, leaf area, leaf length and shorter days to 50% flowering under the Legon environment compared to Bunso. These variations may stem from differences in weather patterns and soil conditions between the two locations [31]. Genotypes that produced significantly more leaves in Bunso were not favoured in Legon. The findings from this research agree with those of earlier studies [13]. In their study, the genotypes assessed showed better yield in Kenya, but the yield was reduced when the same genotypes were planted in Tanzania. Their findings further indicated that the genotypes respond differently at different geographic locations. Similarly, Dinssa et al. [15] indicated that it is unlikely that the same amaranth genotype would consistently exhibit high performance across different locations or seasons, as its performance may vary depending on environmental conditions. Thus, it is important to target selection for a particular environment. Schafleitner et al. [7] also reported variations in leaf yield of amaranth genotypes among different locations and seasons. Amaranth genotypes may have several combinations of characteristics which play an important role in enabling its survival under unfavourable growing environments, including poor soil nutrients, varied temperatures, and soil moisture [31]. Nyasulu et al. and Idowu-Agida et al. [24,32] attributed adaptability of different genotypes to a particular environment to the inherent genetic diversity which resulted in increased chances of survival. The authors noted that to effectively maintain, evaluate, and utilize plant species in breeding and crop improvement programs to obtain better yielding varieties, it is essential to calculate the level of morphological diversity present. Therefore, it is necessary to assess amaranth genotypes across a range of conditions [33,34]. Again, Brammer, [35] establishes that vegetable amaranth thrives well and achieves optimum growth in environments with temperatures above 25 °C. It was also revealed that warm and wet conditions seem favorable for amaranth production as these conditions have an effect on important traits such as plant height and number of branches, which may have impact on leaf yield [24]. These imply that environmental/agricultural management and genetic composition of the amaranth genotypes studied have interrelated effects on the physical expression of some of the traits. The results affirm the findings of Panthee et al. [36] who determined that the growing environment has a major impact on crop production, even while underlying genetic variables cause variation among crop genotypes.

The assessment of phenotypic association among the amaranth genotypes is a key objective of the present study. The grouping of amaranth genotypes based on the cluster analysis revealed a broad genetic diversity among the tested genotypes. This is particularly useful for selection and utilization in future breeding studies to widen the genetic base of amaranth. All the phylogenetic trees grouped the amaranth genotypes into three major clusters. However, each of the clusters consists of distinct members of genotypes. The observed association and distinctiveness based on the cluster analysis confirms the wide variation existing among the studied amaranth genotypes. The cluster variable analysis explains the contribution of individual traits to the observed variation among the amaranth genotypes. According to the results, all the quantitative traits contributed to the existing variations among amaranth genotypes. However, agro-morphological traits such as plant height, leaf area, marketable leaves, chlorophyll content and days to 50% flowering were more informative. Apart from the clustering of amaranth genotypes into various groups, the agro-morphological data was able to reveal group of traits such as plant height, marketable leaves, and days to 50% flowering that contributed significantly to the diversity among the tested genotypes. Oduwaye et al. [26] indicated that variables like leaf and stem characteristics are homogeneous characters that are important in primary selection criteria for vegetative improvement of vegetable amaranth.

Based on the observed high genetic diversity in this research, the genotype evaluated can be selected as potential parent material for breeding trait specific amaranth varieties. Similarly, the diverse traits are essential tools for hybridization to combine desirable traits, genetic mapping for the identification of genes responsible for traits of interest and genomic selection to predict and select best performing individual genotypes. Effective utilization of genetic diversity in the tested amaranth genotypes is a vital requirement for the development of new amaranth varieties that meet the demands of humanity, improving food security, sustainability, and livelihood.

### Correlation among agronomic variables

The positive correlations among traits such as plant height, stem girth, total leaves, number of primary branches, leaf area, leaf width and days to 50% flowering are important indicators of potential genotypes that can be selected and their primary characteristics of interest improved. The strong positive correlations observed between leaf characteristics and number of primary branches implies that branching index (many branches) is a key trait in the exploitation for suitable characteristics. Thus, the production of more branches has an impact on leaf production, the most economic value of vegetable amaranth. In a similar study, Gerrano et al. and Oboh [4,37] noted positive correlations among primary branches, plant height, stem girth, total leaves, and days to 50% flowering. Therefore, it is likely that the more branches a particular genotype has coupled with delayed flowering, the more leaves it may produce. Similarly, the higher the number of leaves, the higher its yield and subsequently the higher income returns to the producer. Therefore, selection of genotypes with high branching index is essential for further improvement.

### Assessment of pests and diseases

The current study noted that leaf rollers/skeletonizer damage averaged 36% to 87.8% at the two locations. However, the reduced incidence at the Legon experimental site may be due to differences in climatic conditions, cropping history, or the prevalence of some insects at specific geographic locations. Appiah-Kubi et al. [11] opined that a host of factors, such as weather conditions, soil type, type of cultivar and cultural practices, contribute to the occurrence and multiplication of diseases in the field. Disease infection on the foliage of vegetable amaranth can reduce its marketable value and negatively affect the economic viability of the crop [11]. In addition, the high severity of disease incidence indicates a reduction in the quantity and quality of the edible leaves. Crop quality, yield, and marketability play important roles in its economic viability; hence, the reduction or loss of these traits due to disease infection compromises the income of farmers. Several species of *Fusarium* have been identified and noted to be responsible for or associated with the fungal disease complex of amaranth [38]. Earlier research has indicated that the occurrence and level of spread of fungal diseases such as *fusarium* rot can be through water and contaminated seeds [5]. Thus, wet rot diseases are influenced by the amount of rainfall and relative humidity, as a result, they are common and severe in areas with heavy rain [39]. This may be the cause of the high incidence of wet rot in the Bunso study area. Appiah-Kubi et al. [11] attributed the presence and severity levels of diseases in most famers’ fields in Amaranth growing areas in Ghana to improper cultural and agronomic practices, including farm sanitation and the use of infected seeds which resulted in a high inoculum density in the environment. Among the genotypes tested in this study, the wide variation in the levels of disease incidence and severity at the two locations is an indication of genetic variability in response to diseases. Genotypes with low pest and disease incidence can be selected and evaluated for their resistance levels for inclusion in plant breeding and crop improvement programs. Amaranth genotypes based on their performances in the current study can make significant contributions to productivity and resistant characteristics and should be included in breeding programs. The focus of systematic breeding programs aiming at improving yield and leaf quality traits of vegetable amaranth would require information regarding the extent of genetic diversity in the quantitative and qualitative traits of the crop. This study revealed useful agronomic diversity in the genotypes. Additionally, host plant resistance is a sustainable pests and diseases management strategy which involves selection, breeding, and deployment of resistant varieties. This study fills an important research gap and provides primary information on the genotypes that can be exploited for the development of resistant amaranth varieties for use in amaranth improvement programs in Ghana.

### Identification of promising genotypes

High leaf production, high branching index, delayed days to flowering, leaf length and marketable leaves are desirable agronomic characteristics for the improvement of vegetable amaranth. The identification of high yielding amaranth genotypes (more leaves) and promising traits is needed to facilitate the efficient utilization of germplasm in breeding programs. Amaranth genotypes that have high yielding characteristics coupled with disease resistance are important requirements for selection [1]. In the present study, yield attributing traits such as mean values of total leaves, number of primary branches, leaf length, and marketable leaves were higher than the improved checks, whiles mean number of days to 50% flowering was delayed compared with the local checks used. Genotype GH10182 had maximum leaf yield (TTL), more primary branches, delayed flowering, more marketable leaf production and minimum disease incidence. This may provide genetic properties useful for the development of high yielding and resistant varieties. Similarly, genotypes GH10281, GH10285, GH10317, GH10284, GH10291, GH10090, GH10193, GH10186, GH10141 and GH10297 can be selected based on their gene specific traits that are desirable to the breeder or farmer.

## Conclusion

Considerable diversity was observed among the tested amaranth genotypes based on the assessment of agro-morphological characteristics and distinct clusters of the phylogenetic trees. The molecular markers used were able to discriminate among the amaranth genotypes to reveal their broader variations based on high values of PIC, gene diversity and Heterozygosity. Generally, pest and disease incidence was higher at the Bunso study than at the Legon study site, genotypes GH10199, GH10182, GH10193 and GH10295 were found to have minimum disease incidence in the Legon study site. Eleven promising trait-specific genotypes; GH10281, GH10182, GH10285, GH10317, GH10284, GH10291, GH10090, GH10193, GH10186, GH10141 and GH10297 were identified for leaf yield, primary branches, and delayed days to 50% flowering. Genotypes GH10281, GH10090, GH10284, GH10317, GH10141 and GH10291 produced ∼1.6 to 27% more leaves than the best check A2002.

## Supporting information

S1-S3 Figs Cluster analysis of (1) quantitative (2) qualitative, and (3) molecular attributes of 21 amaranth genotypes. (DOCX)

S4 Fig Percentage incidence of disease symptoms. (DOCX)

S5 Fig Percentage incidence of insect pests’ population. (DOCX)

S6 Fig Percentage disease incidence and percentage disease severity of amaranth genotypes planted in Bunso and in Legon. (DOCX)

S7 Fig Percentage insect pests’ damaged incidence and percentage severity of insect pests’ damage of amaranth genotypes planted in Bunso and in Legon. (DOCX)

S8 Dataset Dataset on Amaranth, morphological, pest, disease and molecular (ISSR and SSR) experiments. (XLSX)
