# Supplementary material for: Agro-morphological and molecular characterization of Amaranthus genotypes
Source: PLoS One. 2025 Sep 23;20(9):e0328567. doi: 10.1371/journal.pone.0328567 (PMC12456769; doi:10.1371/journal.pone.0328567)
Supplement: S1-S3 Figs — (DOCX) [file pone.0328567.s001.docx]

2

1

3


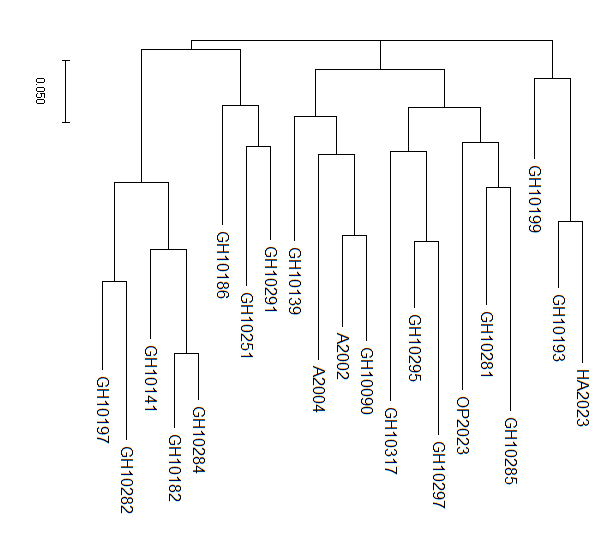

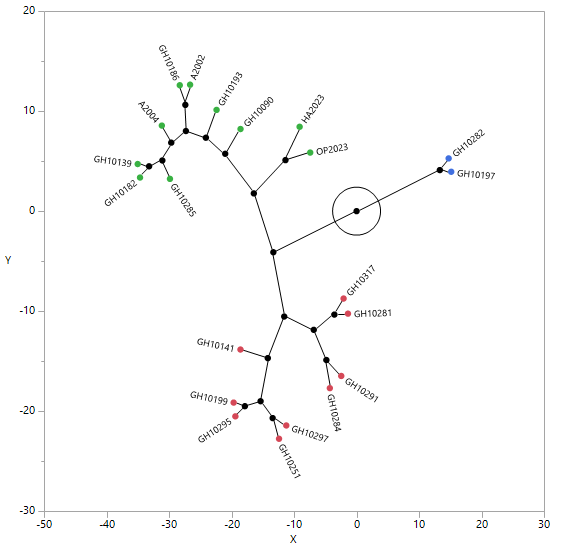

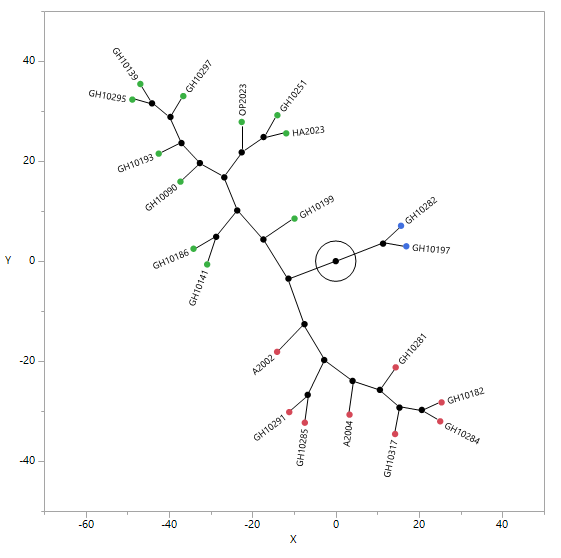


**I**

**III**

**III**

**I**

**II**

**II**

**S1-S3 Figs:** cluster analysis of (1) quantitative (2) qualitative, and (3) molecular attributes of 21 amaranth genotypes
