# Supplementary material for: Agro-morphological and molecular characterization of Amaranthus genotypes
Source: PLoS One. 2025 Sep 23;20(9):e0328567. doi: 10.1371/journal.pone.0328567 (PMC12456769; doi:10.1371/journal.pone.0328567)
Supplement: S4 Fig — (DOCX) [file pone.0328567.s002.docx]

**S4 Fig:** percentage incidence of disease symptoms.
