# Supplementary material for: Agro-morphological and molecular characterization of Amaranthus genotypes
Source: PLoS One. 2025 Sep 23;20(9):e0328567. doi: 10.1371/journal.pone.0328567 (PMC12456769; doi:10.1371/journal.pone.0328567)
Supplement: S5 Fig — (DOCX) [file pone.0328567.s003.docx]

**S5 Fig:** percentage incidence of insect pests’ population.
