# Supplementary material for: Agro-morphological and molecular characterization of Amaranthus genotypes
Source: PLoS One. 2025 Sep 23;20(9):e0328567. doi: 10.1371/journal.pone.0328567 (PMC12456769; doi:10.1371/journal.pone.0328567)
Supplement: S6 Fig — (DOCX) [file pone.0328567.s004.docx]

**S6 Fig:** percentage disease incidence and percentage disease severity of amaranth genotypes planted in Bunso and in Legon
