# Supplementary material for: Agro-morphological and molecular characterization of Amaranthus genotypes
Source: PLoS One. 2025 Sep 23;20(9):e0328567. doi: 10.1371/journal.pone.0328567 (PMC12456769; doi:10.1371/journal.pone.0328567)
Supplement: S7 Fig — (DOCX) [file pone.0328567.s005.docx]

**S7 Fig:** percentage insect pests’ damaged incidence and percentage severity of insect pests’ damage of amaranth genotypes planted in Bunso and in Legon
